# The significance of indirect costs—application to clinical laboratory test economics using computer facilities

**DOI:** 10.1155/S1463924689000374

**Published:** 1989

**Authors:** F. R. Hindriks, A. Bosman, P. F. Rademaker

**Affiliations:** 1 Central Laboratory for Clinical Chemistry University Hospital Groningen Groningen Netherlands Antilles; 2 Department of Management Science and Business Administration of the Economic Faculty University of Groningen Netherlands Antilles

## Abstract

The significance of indirect costs in the cost price calculation of clinical chemistry laboratory tests by way of the production centres method has been investigated. A cost structure model based on the ‘production centres’ method, the Academisch Ziekenhuis Groningen (AZG) 1-2-3 model, is used for the calculation of cost and cost prices as an add-in tool to the spreadsheet program Lotus 1-2-3. The system specifications of the AZG 1-2-3 cost structure model have been extended with facilities to impute all relevant indirect costs to cost centres by aid of allocation rules, which can be chosen freely. The inference is made that as indirect costs play a more important part in decision-making processes concerning planning and control, the specification of the relation to the cost centres should be determined in a more detailed way. The AZG 1-2-3 cost structure model has therefore been extended in order to increase the significance as a management tool for laboratory management.


Journal of Automatic Chemistry Vol. 11, No. 4 (July–August 1989), pp. 174–178

### The significance of indirect costs – application to clinical laboratory test economics using computer facilities

**F. R. Hindriks***

*Central Laboratory for Clinical Chemistry, University Hospital Groningen, Groningen, The Netherlands*

**A. Bosman and P. F. Rademaker**

*Department of Management Science and Business Administration of the Economic Faculty, University of Groningen, The Netherlands*

*The significance of indirect costs in the cost price calculation of clinical chemistry laboratory tests by way of the production centres method has been investigated. A cost structure model based on the 'production centres' method, the Academisch Ziekenhuis Groningen (AZG) 1–2–3 model, is used for the calculation of cost and cost prices as an add-in tool to the spreadsheet program Lotus 1–2–3. The system specifications of the AZG 1–2–3 cost structure model have been extended with facilities to impute all relevant indirect costs to cost centres by aid of allocation rules, which can be chosen freely. The inference is made that as indirect costs play a more important part in decision-making processes concerning planning and control, the specification of the relation to the cost centres should be determined in a more detailed way. The AZG 1–2–3 cost structure model has therefore been extended in order to increase the significance as a management tool for laboratory-management.*

### Introduction

The registration of costs and the calculation of cost prices in health care, especially for laboratory tests, are increasingly important [1–3]. Among other things, interest is focused on computer systems with which all relevant costs, both direct and indirect, can be registered. By means of databases, the costs of the activities of organizational units can be determined beforehand and the cost prices of services to be rendered and the consequences of policy decisions can be judged.

Recently, the Academisch Ziekenhuis Groningen (AZG) 1–2–3 cost structure model has been developed by us as a support for the decision-making concerning the allocation of production factors [4]. In principle, direct costs in addition to indirect costs can be fed into the model. This takes place through an addition method with which the indirect costs are transformed into direct costs. In everyday practice it often appears to be a problem to determine the relation between indirect and direct costs unambiguously.

In this paper, the significance of indirect costs in the overall costs of clinical chemistry tests is examined. Here the question is raised of the way in which the AZG 1–2–3 cost structure model should possibly be adjusted in order to allocate the relevant indirect costs according to the activities, taking into account the nature of the various indirect costs.

### Costs in an organization

In any organization costs are incurred. Costs are defined as an aggregate of applied quantities of production factors multiplied by their prices per unit. Usually costs are subdivided into categories. Thus direct and indirect costs are distinguished in addition to fixed and variable costs. The distinction between direct and indirect costs is very significant for an organization, as thereby a specification is given of the coherence between products, functions and activities existing in any organization. This is a specification that is especially significant for management making decisions on matters of planning and control.

Direct costs are costs that can be directly related to a particular product on the basis of a technical and organizational relationship. Indirect costs do not have this characteristic and usually they can be imputed directly to functions and activities only. They can be imputed (in commercial economics the term used is 'allocated') to products on the basis of allocations which are often arbitrary. The distinction between direct and indirect is first of all dependent on the definition of the term product. Strictly, in cost-price calculation a product is defined as the final products (services) which are marketed by an organization and for which a price is paid and/or for which a remuneration is obtained (e.g. from a giver of grants). In order to effect a good balance of the exchange process possible, the price will have to be compared with the cost-price of the final product. It is obvious that the indirect costs will have been included in the cost-price in one way or another.

In a hospital organization, the diagnoses and therapies are the final products, which are compensated by returns in the form of rates for days of treatment, consultations and operations. The costs which show a direct relation to these final products are direct in nature and those which do not show this characteristic are indirect. Strictly, this means that all costs of departments such as the laboratory, technical department and civil department, are considered to be indirect by the organization of the hospital. This is one of the reasons why the costs and achievements of the laboratory are registered in one auxiliary cost centre in hospital administration.

From the point of view of planning and control, the various services and departments of larger organizations

* To whom correspondence should be addressed.

0309–1902/89 $3.00 © 1989 Taylor & Francis Ltd.

are split into sub-systems, so that each sub-system can be studied separately. The products of most sub-systems are then regarded as achievements (intermediate products), for which there is no direct relation to the return of the organization. In fact, the products of most sub-systems are achievements which contribute to the realization of the quality of the final products of the organization. In order to judge the control of a department or service in hospital organization, it is necessary that beforehand statements can be made on the cost and return to be expected (planning) and that afterwards it can be checked whether the control was either good or bad. Therefore, figures on the total cost and return per department or service beforehand and afterwards are necessary. The mutual supplies of achievements among the sub-systems of an organization should therefore be registered by means of internal imputation systems. The way in which this takes place, particularly the extent of specification, determines the way in which control can be judged.

### Allocation of costs

By means of the costs a cost-price per product, service or achievement can be calculated. By cost-price, the amount of direct and indirect costs per product unit is meant.

There are various methods for the cost-price calculation of final products. Two examples, of which the second is suitable for planning, are the following.

*Primitive addition method.* The indirect costs are imputed to the separate products in the form of an addition to (part of) the direct costs.

*Production centres method.* According to this method, the various costs are imputed to the various products on the basis of the relation of the application of production factors which caused the costs for the realization of the products. It should be investigated how far achievements, coming from different organizational sub-systems, were necessary for each product and therefore have to be considered as an indispendable 'derived' production factor. With this information, allocations can be determined.

The allocation of costs through the production centres method assumes an organization in which various functions with the costs connected to them are distinguished, which can be imputed to other functions or products. These functions are called cost centres and the final products are called cost bearers.

Auxiliary cost centres are cost centres that supply achievements, not final products. The costs of auxiliary cost centres are imputed to other auxiliary or main cost centres.

Main cost centres are the cost centres of which the achievements directly benefit the final products, the cost bearers. The production centres method, which forms the basis for the AZG 1–2–3 cost structure model, is the method of cost price calculation in which the coherence

*Table 1. Supplies of services to and within our laboratory entailing indirect costs and stating the share in the total cost of laboratory tests.*

| | Share (%) |
|---|---|
| 1. Sample collection | 5 |
| 2. Sample transportation | <1 |
| 3. Preparation of work and distribution | 3 |
| 4. General services | 3 |
| 5. Service and maintenance | 6 |
| 6. Computer system | 5 |
| 7. Management and supervision | 4 |
| 8. Administration | 2 |
| 9. Training, education and research | 6 |
| 10. Housing | 5 |
| 11. Scientific staff | 2 |
| 12. Laundry | <1 |
| Total share of indirect costs | 40 |

Note: one part of the indirect costs has not been included in this set-up, i.e. hospital management, and another part, housing and scientific staff, is probably too low in this estimation.

between the achievements and final products are explicitly laid down. This coherence in a way represents the production structure of an organization and it is essentially important in judging the control.

### Indirect costs

Many mutual supplies of both external and internal achievements to which indirect costs are entailed can be distinguished in the clinical chemistry laboratory. An inventory of services and the indirect costs entailed in our laboratory are stated in table 1.

The question to be asked now is which indirect costs are relevant to be involved in the cost price calculation. For this purpose the relation to the products, the results of laboratory analysis should be determined. Cost-price calculation is applied in organizations to support the many decisions that are made, where costs and cost-prices are used as a criterion.

The need to consider indirect costs in cost price calculation is based on a number of reasons:

1. From the planning point of view, it is necessary to judge alternative application possibilities of production factors through which changes in the conduct of business and organization of the mutual supplies result. In the clinical laboratory the sample collection, transportation, reception and preparation of work come into consideration here. However, the organization of the warehouse, the cleaning room and the administration are also important.

2. Costs are used to make statements on the efficiency of an organization and as a means to control efficiency, e.g. by budgetting. By efficiency is meant the extent to which an organization is capable to achieve a certain goal with the given means, this goal often being a certain rate of production. In order to make statements on this, the cost function will have to be divided into the production functions lying behind it. As achievements to which indirect costs are entailed contribute considerably to the realization and quality of the products, it is important to include the indirect costs in the calculation of cost, especially in order to be able to determine if the supply meets the desired specifications of efficiency and quality, and if the compensation obtained for it is sufficient.

3. In the development of functional budgetting, the significance of know-how concerning the cost of laboratory tests is increasing because alternative application possibilities of the clinical laboratory can favourably affect the allocation of the budgetted production factors within the hospital organisation. In the near future the clinical laboratories will be faced more emphatically with the question of utilization of analytical equipment and staff. Considerations of the capacity to be put into service are affected by the possibilities of having activities executed by external laboratories or by attracting research from outside the organization in order to realize a more favourable rate of utilization. For both situations it is important to know all relevant costs for laboratory tests.

For a judgement by the management, these three reasons have to be stated with the relevant arguments, which is why indirect costs cannot be excluded from consideration.

Usually it is difficult to transform indirect costs into direct costs, as a direct relation with the results of analysis cannot be derived. The indirect costs specified in table 1 comprise a considerable part of the total costs of our laboratory (at least 40%). With this the ratio of direct and indirect costs is 3:2.

### The AZG 1–2–3 cost structure model and indirect costs

In the original version of the AZG 1–2–3 cost structure model, indirect costs, in as far as this is relevant and realizable, can be transformed into direct costs or be imputed to the main cost centres by way of a variant of the primitive addition method. This method is based on the assumption that the ratio of indirect costs (overhead) to the total cost is equal for every sort of activity, and corresponds to the ratio of total indirect costs to total cost. In everyday practice, it can be questioned whether this assumption is correct. Should this not be the case, the rule used in the primitive addition method has to be specified further. Such rules are called allocation rules.

An allocation rule consists of quantities or factors by means of which indirect costs can be split up. They are rules that specify the relations between the achievements which were made and the production of final products or achievements. The factors included in these rules will differ according to the nature of the existing relations and the desired accuracy with which they are described. These factors are expressed in physical units of the achievement to be made (e.g. the number of kilowatt hours in electricity). Some examples of these factors are the number of tests, direct costs per activity, surface measure and number of employees.

Per auxiliary cost centre, one or more allocation rules are specified by which the costs can be allocated. Then an allocation will result.

The indirect costs are imputed by way of the following equation:

$$k_{ij} = K_{Ji}.[x_{ij}/\Sigma(x_{i1} \ldots x_{im})]$$

where $k_{ij}$ = the indirect costs of the auxiliary cost centre $i$ which is imputed to the product $j$; $i_{i,j} = 1 \ldots m$; $K_{Ji}$ = the total cost of the achievement $i$; and $x_{im}$ = the technical factor of achievement $i$ for the specified products from 1 up to and including $m$ series.

The distribution of indirect costs into the various activities by means of allocations have thus been included in the AZG 1–2–3 cost structure model.

The AZG 1–2–3 application program, based on the Lotus 1–2–3 program, has been extended with facilities to distribute indirect costs over various activities by allocations known or to be chosen. The program can be applied on any IBM-compatible microcomputer with an internal memory of at least 384 kB, DOS operating system and using the Lotus 1–2–3 program.

### Examples of application

To illustrate the allocation of indirect costs, the cost of housing and computer usage will serve as examples.

### *Housing*

As a rule it is generally accepted that the cost of housing is allocated by the ratio of the number of net square metres. The price per square metre is determined by

$$p_{Ji} = K_{Ji}/\text{total number of square metres}$$

where $p_{Ji}$ = the cost-price of achievement $i$ (in this case housing).

For the calculation of the contribution of indirect costs of housing to a certain achievement $j$, the number of net square metres that has to be hired for the production of $j$ should be determined. A relation between the production of $j$ and the number of achievements of $i$ in the case of cost of housing usually does not exist. In order to produce the achievement, $j$ square metres are needed. This number will not flucutuate with the real production of $j$. Mostly an amount is calculated that consists of $p_{Ji}$ times the number of square metres needed.

### *Computer costs*

An example showing detailed figures of the imputation of indirect costs of computer usage to the achievements of the SMAC automated analyser (Technicon Instruments,

*Table 2. Source data with relation to the achievements of the hospital computer.*

| | Hospital computer achievements | | | |
|---|---|---|---|---|
| | Total | CKCL* share (%) | Costs according to system of tariffs† | |
| CPU time | 468 037 437 | 15·2 | × Dfl 11·E-4 = Dfl | 78 256 |
| Disc accesses | 1 568 052 673 | 17·9 | × Dfl 35.E-4 = Dfl | 928 385 |
| SPOOL records | 163 760 612 | 19·1 | × Dfl 30.E-4 = Dfl | 93 835 |
| Total | | | Dfl | 1 154 476 |

* Abbreviation of our laboratory.
† Dfl = Dutch guilders.

*Table 3. Source data with relation the achievements of the SMAC analyser.*

| | CKCL achievements | |
|---|---|---|
| | Total | SMAC share (%) |
| Tests | 2 895 314 | 55·3 |
| Requests | 425 681 | 31·3 |
| Direct costs (Dfl) | 8 285 600 | 11·3 |

*Table 4. Results of imputing costs of hospital computer to the SMAC analyser in three different ways (all amounts in guilders).*

| | | Production centres method | |
|---|---|---|---|
| | Addition method | Allocation rule | Technical coefficient |
| Per request | 0·98 | 2·71 | 2·93 |
| Per test | 0·08 | 0·40 | 0·24 |
| Imputation | 130 456 | 361 350 | 390 944 |

Tarrytown, NY, USA) illustrates the significance of the imputation of indirect costs in case there exists a direct relation between the production of $j$ and the number of achievements of $i$.

It can easily be seen that there is a relation between the SMAC production (number of tests or requests) and the application of the hospital computer (in the form of CPU time, disc accesses, SPOOL records) as a production factor. It is relatively easy (though time consuming) to determine the amount taken up by SMAC through the account data of the computer system (see table 2). It will be more difficult to define the achievement unit of the computer system, the system second, and to calculate a price per unit for it, as the specifications lying behind it are very arbitrary.

In practice, computer costs are often allocated through a tariff system, for which it is not clear in what way it was specified, as a result of which gross inaccuracies can result. As the decisions that are made partly on the basis of the results of allocation of costs are considered more important, the imputation of costs should take place in a more detailed (and hence more accurate) way. In such a case an application of the indirect costs on the basis of a simple addition method will not suffice; instead, taking into consideration the relation with the production of $j$, the application of the computer will have to be determined as a technical coefficient, and the costs entailed to be considered direct.

In tables 2 and 3, the source data with respect of the achievements of the hospital computer and the SMAC analyser in our laboratory are stated. In various ways the indirect costs for the use of the hospital computer can be imputed, the results of which are stated in table 4. The calculations are given in the Appendix. Note that £1 corresponds with about Dfl 3.70.

The results show that large differences in the imputation arise and that it is essential to recognize them as such and to realize that these differences are strongly dependent on the chosen (arbitrary) rules lying behind it. In practice, it is often chosen as a starting point to impute indirect costs on a high aggregation level as the difficult specification is not always necessary. As the decisions based on that are considered more important, the necessity of specifying the relations concerned in more detail will increase.

The primitive addition method takes these computer costs as fixed and distributes these costs to the laboratory products (requests, tests) randomly, not based on any specification of the relation between computer achievements and laboratory products. The method can impute these costs after they have been made.

The production centres method imputes costs according to the specification of the relation between the computer achievements and the laboratory production process, which gives results closer to reality than the primitive addition method.

The imputation through technical coefficients is time consuming because of the detailed specification of the workload, i.e. the definition of the unit of computer achievement, the system second and the relation between the system second and the laboratory products. A great advantage for reasons of planning and control, is to use this method beforehand. This method can be considered as a reference method.

The use of allocation rules is simple and quick to perform. It seems to be the second-best method and for many applications gives relevant information sufficient to make the right decisions.

### List of definitions

*Activity*: A description of a technical transformation process that for certain reasons can be considered as a unit and that results in the production of final products or intermediate products.

*Allocation*: Allocation of the indirect costs which an auxiliary cost centre imputes to a number of main cost centres.

*Allocation rule*: An allocation rule consists of quantities or factors by means of which the indirect costs of an auxiliary cost centre can be allocated to specified activities.

*Cost*: Aggregate of production factors used multiplied by their prices per unit to produce a certain production quantity.

*Cost centre*: An organization unit, in which all costs, with reference to certain activities, are specified. See also Activity.

*Direct and indirect costs*: Direct costs are costs that can be related directly to a particular final product on the basis of a technical and organizational relation. Indirect costs do not have this characteristic, but they can usually be directly imputed to functions and activities on the basis of causal relations.

*Final product*: The output of an activity which is sold by the organization and to which the revenues are directly related.

*Intermediate product (achievement)*: Intermediates or intermediate products are the output of these activities that are not sold by the organization but which are necessary to produce the final products.

*Occupancy*: Indicates which portion of the availability capacity of production factors is utilized.

*Production factor*: Is applied for the sake of activities in realizing (final) products.

*Production quantity*: Number of units of an activity produced in a certain time period.

*Rate of application*: The use of a production factor per unit time in order to achieve a certain rate of production.

### Acknowledgement

The authors are grateful to W. Liet for his investigation of the relation between the costs of using the hospital computer and the activities of the laboratory. Some of his findings have been used in this study.

### Appendix

Imputing the costs of the hospital computer to the SMAC analyser in three different ways:

*1. The primitive addition method*

The share of SMAC in the total CKCL costs is 11·3%; in total Dfl 1 154 476 is imputed to the laboratory. The addition for SMAC is then 11·3% of Dfl 1 154 476 or in total Dfl 130 456, i.e. Dfl 0·98 per request or Dfl 0·08 per test.

*2. Allocation through allocation rules (production centres method)*

The indirect costs of computer usage are allocated by the ratio of the number of tests or in a given case the number of requests into the activities of SMAC. The share of SMAC in the total number of CKCL tests or requests is 55·3% and 31·3%, respectively. Imputation to SMAC through 55·3% of Dfl 1 154 476 over 1 601 136 tests will then result in an addition of Dfl 0·40 per test or through 31·3% of Dfl 1 154 476 over 133 428 requests in Dfl 2·71 per request.

*3. Through technical coefficients (production centres method)*

There is a relation between the production of the SMAC activity and the achievements of the hospital information system, which can be indicated by the determination of a technical coefficient.

The hospital computer production factor is expressed in the physical unit of the system second (syssec) and has been defined as follows:

$$1 \text{ unit} = 0{\cdot}222 \text{ CPU time} + 0{\cdot}688 \text{ disc access} + 0{\cdot}09 \text{ SPOOL record}$$

The price per unit of the syssec amounts to Dfl 29.E-4 according to the system of tariffs.

The technical coefficient of the hospital computer production factor for SMAC activity amounts to 1005 syssec, i.e. dealing with (producing) one request by SMAC takes up 1005 syssec of the hospital information system.

Because of the relation between production and supplied achievement, the costs of hospital computer usage are considered direct by the SMAC analyser. The eventual addition will result in Dfl 2·93 per request or Dfl 0·24 per test.

### References

1. Krieg, A. F., Israel, M., Fink, R. and Shearer, K. L., *American Journal of Clinical Pathology*, **69** (1978), 525.
2. Broughton, P. M. G. and Hogan, T. C., *Annals of Clinical Biochemistry*, **1** (1981), 330.
3. Stilwell, J. A., *Journal of Clinical Pathology*, **34** (1981), 589.
4. Hindriks, F. R., van der Slik, W., Bosman, A., Frowein, Chr. and Kamps, H., *Journal of Automatic Chemistry*, **4** (1986), 178.

